# Exploration of the Graphene Quantum Dots-Blue Light Combination: A Promising Treatment against Bacterial Infection

**DOI:** 10.3390/ijms25158033

**Published:** 2024-07-23

**Authors:** Roberto Rosato, Giulia Santarelli, Alberto Augello, Giordano Perini, Marco De Spirito, Maurizio Sanguinetti, Massimiliano Papi, Flavio De Maio

**Affiliations:** 1 Department of Basic Biotechnological Sciences, Intensive and Perioperative Clinics, Università Cattolica del Sacro Cuore, 00168 Rome, Italy; 2 Dipartimento di Neuroscienze, Università Cattolica del Sacro Cuore, Largo Francesco Vito 1, 00168 Roma, Italy; 3 Department of Laboratory and Infectious Sciences, Fondazione Policlinico Universitario A. Gemelli IRCCS, 00168 Rome, Italy

**Keywords:** Graphene Quantum Dots, blue light, antimicrobial treatments

## Abstract

Graphene Quantum Dots (GQDs) have shown the potential for antimicrobial photodynamic treatment, due to their particular physicochemical properties. Here, we investigated the activity of three differently functionalized GQDs—Blue Luminescent GQDs (L-GQDs), Aminated GQDs (NH_2_-GQDs), and Carboxylated GQDs (COOH-GQDs)—against *E. coli*. GQDs were administrated to bacterial suspensions that were treated with blue light. Antibacterial activity was evaluated by measuring colony forming units (CFUs) and metabolic activities, as well as reactive oxygen species stimulation (ROS). GQD cytotoxicity was then assessed on human colorectal adenocarcinoma cells (Caco-2), before setting in an in vitro infection model. Each GQD exhibits antibacterial activity inducing ROS and impairing bacterial metabolism without significantly affecting cell morphology. GQD activity was dependent on time of exposure to blue light. Finally, GQDs were able to reduce *E. coli* burden in infected Caco-2 cells, acting not only in the extracellular milieu but perturbating the eukaryotic cell membrane, enhancing antibiotic internalization. Our findings demonstrate that GQDs combined with blue light stimulation, due to photodynamic properties, have a promising antibacterial activity against *E. coli*. Nevertheless, we explored their action mechanism and toxicity on epithelial cells, fixing and standardizing these infection models.

## 1. Introduction

In the ongoing effort to combat antibiotic resistance, there is a growing focus and research on novel nanomaterials that hold promise against several human pathogens.

Graphene Quantum Dots (GQDs) are graphene structures characterized by a two-dimensional transverse dimension between 10 and 100 nm, exhibiting remarkable chemical, physical, and biological properties. GQDs consist of a monolayer of carbon atoms, yet the synthesized majority often include oxygen and hydrogen functional groups [1].

In contrast to traditional inorganic quantum dots, GQDs offer numerous advantages such as facile synthesis, robust fluorescence, resistance to photobleaching, exceptional solubility and biocompatibility [2,3]. Due to these properties, GQDs have demonstrated significant promise across various applications including bioimaging, drug delivery, and biosensing [4,5]. The exploration of GQDs’ antimicrobial properties represents a significant advancement in nanotechnology-based therapeutic strategies, offering innovative solutions to the growing challenge of antibiotic resistance. Indeed, recent studies have shown that GQDs can target a wide range of microorganisms, including antibiotic-resistant bacteria and pathogenic fungi, highlighting their potential as versatile antimicrobial agents [3,6,7]. Most of the GQDs’ antimicrobial properties derived from the surface functional groups: while amino-functionalized GQDs increases cell entry and drug delivery, carboxylated GQDs showing ability to strongly interact with positive charges were proposed for molecular targeting [3].

Of note, the recent discoveries which have revealed an additional function of GQDs in generating singlet oxygen (^1^O_2_) under light exposure, thus indicating their potential as candidates for photodynamic therapy (PDT) [8,9,10]. GQDs obtained through electrochemical means, when exposed to blue light (470 nm), produced ^1^O_2_ and various reactive oxygen species, resulting in oxidative stress and the death of human glioma cells [11,12].

During a PDT, the photosensitizers, upon exposure to light irradiation of specific wavelengths (470 nm), initiates the generation of reactive oxygen species (ROS) within its immediate proximity, thereby eliciting a targeted biological response in the affected tissue [13,14].

Antimicrobial PDT may represent a particularly promising alternative treatment for multidrug-resistant microbes [15,16].

The ability to generate ^1^O_2_ and various reactive oxygen species allows GQDs to effectively disrupt microbial cell walls and inhibit biofilm formation [17,18]. However, despite the demonstrated efficacy of these materials, the specific impact of GQDs on bacteria remains largely unexplored, suggesting potential opportunities for further investigation.

Here, we investigate the photodynamic antibacterial properties of GQDs, particularly focusing on their interaction with *Escherichia coli* (*E. coli*), a Gram-negative bacterium commonly found in the human intestinal microbiota being capable of causing a spectrum of infections ranging from mild gastroenteritis to life-threatening conditions such as urinary tract infections and sepsis [19]. Our study delves into assessing the antimicrobial potential of GQDs against *E. coli*, leveraging their inherent antibacterial activity induced upon exposure to irradiation.

## 2. Results

### 2.1. Stimulation of Graphene Quantum Dots Enhances Their Antibacterial Activity against E. coli

**Figure 1:**
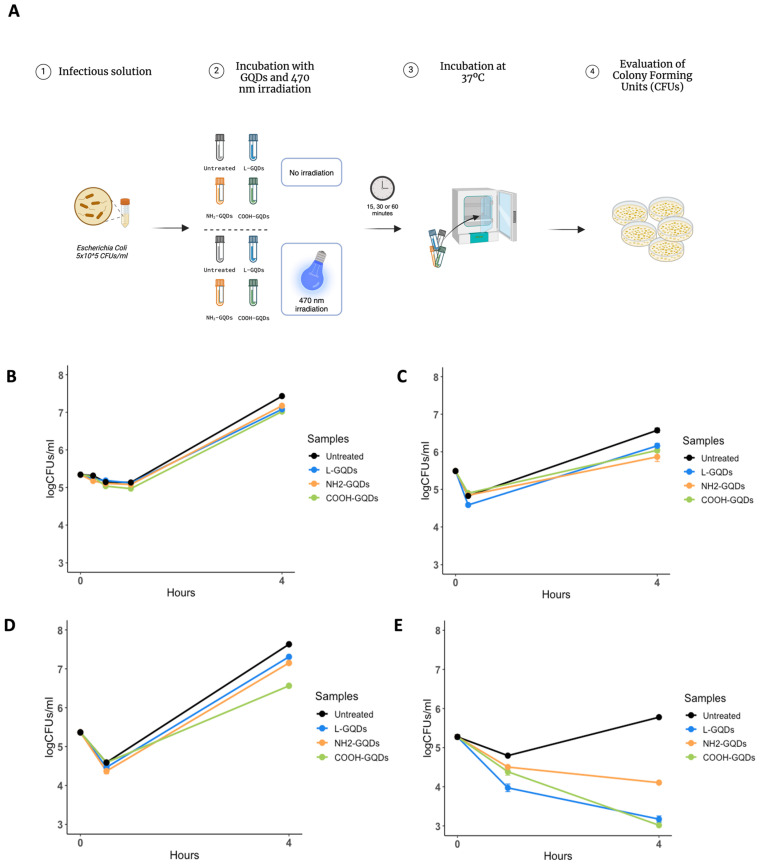
Graphene Quantum Dots irradiated with blue light significantly reduce *E. coli* viability. Schematic representation of the experimental model used to assess Graphene Quantum Dots (GQDs) activity against *E. coli*. Differently functionalized GQDs (L-GQDs, NH_2_-GQDs and COOH-GQDs), stimulated or not with blue light (470 nm wavelength), were used (**A**). A suspension of *E. coli* at the final concentration of 5 × 10^5^ CFUs/mL was incubated with a suspension of 200 µg/mL GQDs and colonies forming units (CFUs) were measured at 15, 30, 60 min and 4 h later (**B**). A suspension of *E. coli* and GQDs was irradiated with blue light for 15 (**C**), 30 (**D**) and 60 min (**E**) until CFU evaluation 4 h later. CFUs/mL were reported in log10 scale.

To date, several studies provided elusive information about quantum dots, and more generally nanomaterials, antibacterial properties due to numerous and different experimental settings. While the antibacterial effects of the Carbon Quantum Dots were demonstrated without the need for light or other stimuli, Graphene Quantum Dots (GQDs) antibacterial activity appeared associated with blue light stimulation [20]. For this reason, to evaluate the direct antibacterial activity of GQDs, we administrated differentially functionalized GQDs (L-GQDs, NH_2_-GQDs and COOH-GQDs), at the final concentration of 200 µg/mL, to 5 × 10^5^ *E. coli* suspension. This suspension was incubated at standard culture conditions or was irradiated early with blue light (470 nm wavelength) for 15, 30 or 60 min before incubation. Antibacterial activity was measured by counting colony forming units (CFUs) after the stimulations and 4 h later (Figure 1A). The administration of GQDs alone without stimulation did not reduce the bacterial burden (*p* > 0.05) (Figure 1B). Conversely, GQDs’ antibacterial activity was enhanced by blue light and appeared dependent on the time of irradiation (Figure 1C–E). Comparable antibacterial activity was observed when CFUs were measured following 15 and 30 min of stimulation, reducing the *E. coli* growth by 1 log_10_ (*p* < 0.01) for L-GQDs, NH_2_-GQDs and COOH-GQDs treated samples. Intriguingly, untreated stimulated samples showed similar CFUs, suggesting a minimal blue light dependent antibacterial activity. However, when the suspension was exposed to blue light for 60 min, a large decrease was detected with L-GQDs recognized as the most efficient nanomaterial (*p* < 0.01). Moreover, we included an additional step to our experimental model in comparison with previous studies [14,21,22], assessing CFUs 4 h after stimulation. We highlighted that 15 and 30 min of blue light stimulation were not sufficient to mainly inhibit microbial growth (*p* > 0.05). On the contrary, 60 min of blue light irradiation enhanced the antibacterial activity of all tested GQDs compared to untreated sample. L-GQDs and COOH-GQDs appeared to mostly reduce *E. coli* growth (approximately 2 log_10_, *p* < 0.01), whereas NH_2_-GQDs showed a slight but not significant variation in the CFUs count after 4 h of incubation compared to 60 min counting (*p* > 0.05). Despite of studies that assayed the impact of blue light stimulation on GQDs properties, their antibacterial activity was not yet assessed later than 15 min of incubation [14,21].

**Figure 2:**
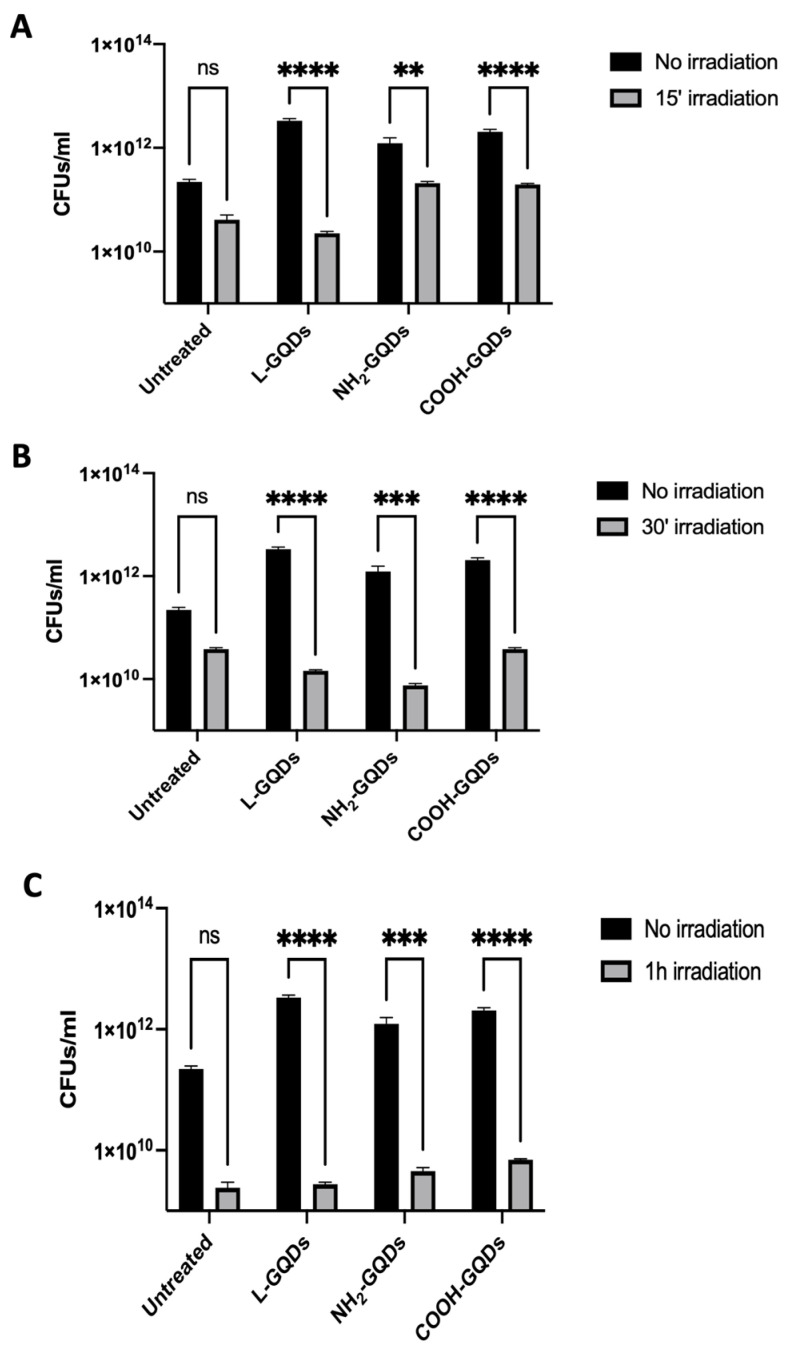
Irradiated Graphene Quantum Dots reduce *E. coli* viability 24 h post stimulation. A suspension of *E. coli* at the final concentration of 5 × 10^5^ CFUs/mL was incubated with 200 µg/mL of differently functionalized Graphene Quantum Dots (L-GQDs, NH_2_-GQDs and COOH-GQDs) alone or irradiated with blue light (470 nm) for 15 (**A**), 30 (**B**) and 60 min (**C**). Colony forming units (CFUs) were assessed 24 h post treatment to measure long-term activity of nanomaterials. CFUs were represented in log10 scale as bar plots. (** *p* value < 0.01; *** *p* value < 0.001; **** *p* value < 0.0001; ns = not significant).

To demonstrate that the antimicrobial effect mediated by GQDs was not transient and immediately evidenced only after stimulation or in the medium-term period (4 h), we performed the same experiment evaluating CFUs 24 h later. As showed in Figure 2, stimulated GQDs were able to preserve the antibacterial activity until 24 h following treatment and blue light stimulation. The major reduction was observed again when GQDs were exposed to blue light for 60 min (Figure 2C), In particular, L-GQDs and COOH-GQDs were able to reduce the bacterial burden by 3 log_10_, whereas NH_2_-GQDs showed a 2 log_10_ decrease. Log_10_ differences between irradiated and not irradiated samples were also summarized in Supplementary Table S1.

These data highlighted that GQDs’ antimicrobial activity against *E. coli* was directly dependent on their irradiation and slightly related to their functionalization.

### 2.2. Stimulated Graphene Quantum Dots Impair E. coli Metabolic Activity

**Figure 3:**
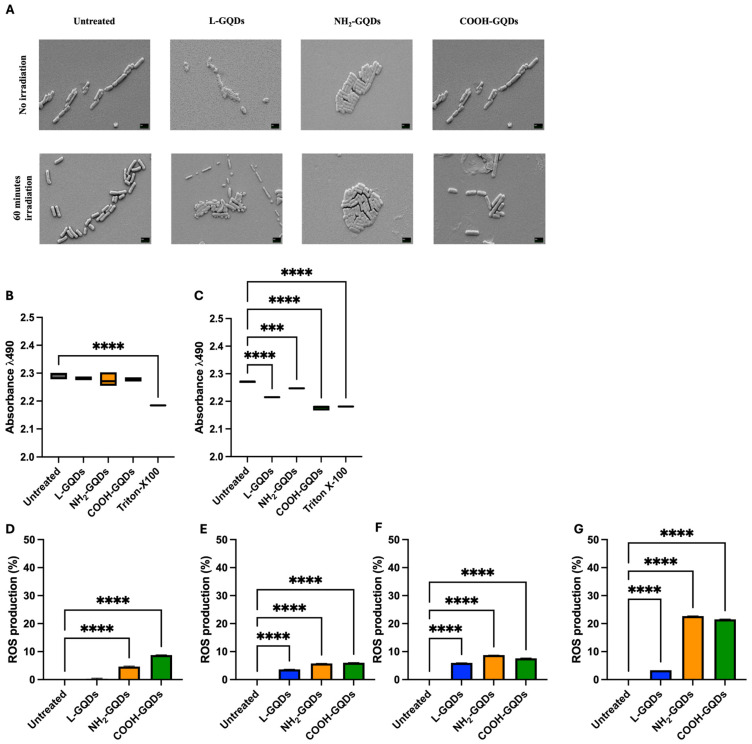
Graphene Quantum Dots do not impair *E. coli* morphology but do affect its metabolic activity and ROS generation. To investigate direct macroscopic activity of Graphene Quantum Dots on *E. coli*, scanning electron microscopy (SEM) was performed on a suspension of *E. coli* at the final concentration of 5 × 10^5^ CFUs/mL treated with 200 µg/mL GQDs (L-GQDs, NH_2_-GQDs and COOH-GQDs), irradiated with blue light (470 nm) for 60 min or not irradiated. Additionally, *E. coli* treated with gentamicin and kanamycin were included as controls. SEM acquisition (magnification: 15,000×, scale bar: 2 µm) was performed 4 h post treatment (**A**). The metabolic effect of GQDs was investigated on a 5 × 10^5^ *E. coli* suspension using the MTS assay. The bacterial suspension was seeded in a 96-well plate and treated with 200 µg/mL GQDs (L-GQDs, NH2-GQDs and COOH-GQDs). Each condition was left non-irradiated (**B**) or blue light irradiated for 60 min (**C**). Untreated bacterial suspension and treated with 2% Triton X-100 were used as negative and positive controls, respectively. Metabolic activity was determined by measuring absorbance (490 nm wavelength) using a Cytation instrument following the MTS assay. *E. coli* cells (5 × 10^5^ CFU/mL) were treated with 200 µg/mL GQDs and either exposed or not exposed to blue light (470 nm) for 60 min to investigate ROS production. ROS levels were measured immediately in a non-stimulated condition (**D**) and after light exposure (**E**). Additional measurements of ROS levels were performed after a 4 h incubation at 37 °C for both the non-stimulated (**F**) and 60 min blue light-stimulated conditions (**G**). Cells were incubated with 20 µM DCFDA at 37 °C for 30 min in the dark prior to fluorescence measurement. Fluorescence intensity was recorded using the Cytation 5 instrument at excitation and emission wavelengths of 485 nm and 535 nm, respectively. (*** *p* value < 0.001, **** *p* value < 0.0001).

Carbon-based nanomaterials have been demonstrated to have a broad-spectrum of antibacterial activities and so have been preferred to develop new strategies against drug-resistant bacteria [23,24]. We have the exploited antibacterial property of graphene oxide and of GQDs against mycobacteria, demonstrating their different activity and impact [3,25,26]. In this context, we investigate the effects of GQDs, stimulated or not with blue light, against *E. coli* by scanning electron microscopy (SEM) analysis and assessing its viability by using the MTS assay, which directly measures its viability. We administrated L-GQDs, NH_2_-GQDs, and COOH-GQDs to a bacterial suspension and exposed it to blue light for 60 min. Untreated sample and samples treated with gentamicin and kanamycin were used as controls. SEM analysis revealed no significant macroscopic changes in the bacterial morphology induced by GQDs compared to the untreated specimens (Figure 2). Furthermore, no changes were revealed between differently functionalized GQDs, while as expected a high decrease in bacterial burden and cell damage was associated with antibiotic treatments. These results suggest that neither GQDs nor blue light stimulation significantly impact on macroscopic features of the bacterial cells. We then seeded *E. coli* treated as previously described in a 96-well plate. The plate was directly incubated (Figure 3B) or treated with blue light for 60 min (Figure 3C). As controls were included a treatment with 2% Triton X-100 treatment (rather than gentamicin and kanamycin) and untreated bacterial solution, respectively. MTS was added and absorbance (λ = 490 nm) was measured using a spectrophotometer. Treatments with L-GQDs, NH_2_-GQDs and COOH-GQDs showed no significant variations in the metabolic activity of *E. coli*, showing slight changes compared to the control (*p* > 0.05). Conversely, the use of Triton X-100 as a control showed a significant reduction (*p* < 0.01). However, exposure to blue light for 60 min resulted in a statistically significant modulation of *E. coli* metabolic activity across all GQDs treatments (*p* < 0.01). Specifically, both L-GQDs, NH_2_-GQDs and COOH-GQDs showed a decrease, reaching values up to Triton X-100 or antibiotic treatments. These results highlight a key role of stimulated GQDs in modulating *E. coli* metabolic processes impairing its viability.

As previously mentioned GQDs induce reactive oxygen species, a property that appears shared from other carbon-based nanomaterials such as graphene oxide [27,28]. Additionally, GQDs, when exposed to blue light (470 nm), increased reactive oxygen species production, resulting in inducing cell death due to oxidative stress [11,12]. For this reason, we finally measured ROS production in *E. coli* culture treated with L-GQDs, NH_2_-GQDs, and COOH-GQDs, as previously described, and exposed to 470 nm wavelength light. ROS production appeared comparable between samples exposed or not to blue light in the first 60 min, when NH_2_-GQDs, and COOH-GQDs were administrated (Figure 3D,E). L-GQDs ROS stimulation was directly linked to light stimulation (Figure 3E). Interestingly, 4 h later, samples treated with NH_2_-GQDs, and COOH-GQDs and exposed to 470 nm blue light showed higher ROS production compared to control and L-GQDS or to counterparts not light exposed (Figure 3F,G). These results corroborated what observed with the MTS assay and demonstrated that GQDs’ antibacterial activity was light dependent and ROS mediated.

### 2.3. Graphene Quantum Dots Exert Extracellular Antibacterial Activity during Infection of Epithelial Cells

**Figure 4:**
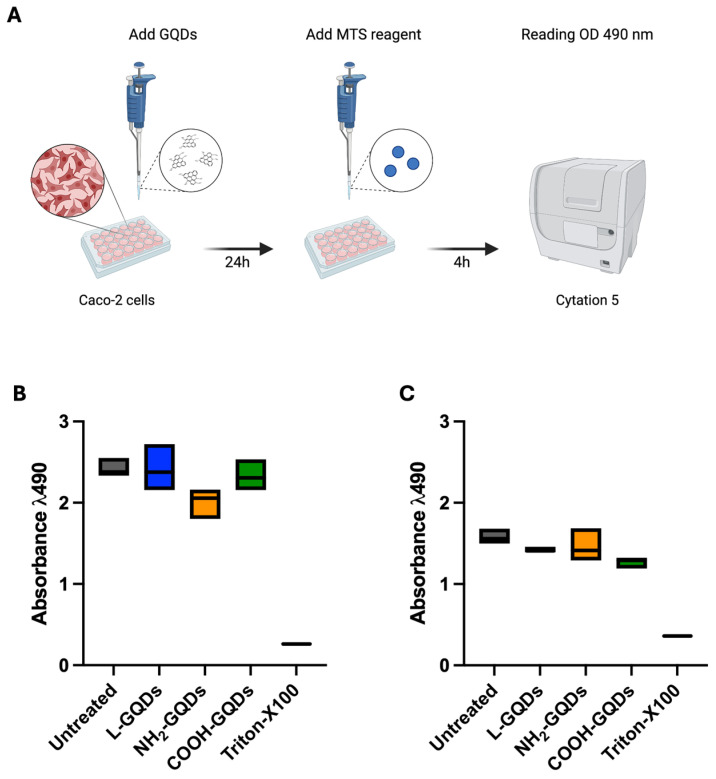
Graphene Quantum Dots exhibited no cytotoxicity on human epithelial cells. Graphene Quantum Dots (GQDs)’s cytotoxicity was assessed on the human colorectal adenocarcinoma cell line (Caco-2) by using the MTS assay (**A**). Caco-2 cells were seeded in a 96 well plate. When the 90% cell monolayer was reached, cells were treated with 200 µg/mL GQDs (L-GQDs, NH_2_-GQDs and COOH-GQDs) and stimulated with blue light for 60 min. Untreated cells and cells treated with 2% Triton X-100 were used as negative and positive controls, respectively. Cytotoxicity was determined by measuring absorbance (490 nm wavelength) using Cytation instrument following the MTS assay at 60 min (**B**) and 4 h (**C**) post treatments.

**Figure 5:**
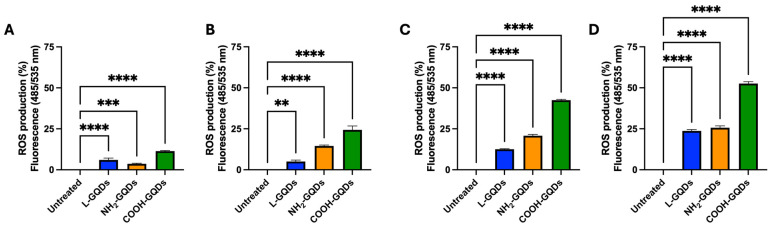
Graphene Quantum Dots enhance ROS generation in in vitro Caco-2 cells model. Caco-2 cells (1.2 × 10^6^ cells/well) were seeded in a 96 wells plate. When the 90% cell monolayer was reached, cells were treated with 200 µg/mL GQDs (L-GQDs, NH_2_-GQDs and COOH-GQDs) and either exposed or not exposed to blue light (470 nm) for 60 min to investigate ROS production. ROS levels were measured in a non-stimulated condition (**A**) and immediately after light exposure (**B**). Additional measurements of ROS levels were performed after a 4 h incubation at 37 °C for both the non-stimulated (**C**) and 60 min blue light-stimulated conditions (**D**). Cells were incubated with 20 µM DCFDA at 37 °C for 30 min in the dark prior to fluorescence measurement. Fluorescence intensity was recorded using the Cytation 5 instrument at excitation and emission wavelengths of 485 nm and 535 nm, respectively. (** *p* value < 0.01; *** *p* value < 0.001, **** *p* value < 0.0001).

We have previously assessed GQD cytotoxicity on macrophages and human peripheral mononuclear blood cells, so that according to the previously described experimental schemes we tested GQDs toxicity on human colorectal adenocarcinoma cells (Caco-2) (Figure 4A). Caco-2 cells were firstly incubated with L-GQDs, NH_2_-GQDs and COOH-GQDs and then stimulated with blue light irradiation (60 min) or not. Cell viability was measured 4 h later. The MTS assay resulted in no statistically significant differences in cell viability when nanomaterials were only administrated to epithelial cells (Figure 4B). Similarly, when GQDs were administrated and stimulated with blue light did not induce cell death in comparison with the untreated cells (Figure 4C). Furthermore, we evaluated reactive oxygen species production after GQDs treatment and blue light irradiation. As shown in Figure 5, administration of GQDs stimulated minimal ROS production after 60 min and 4 h: while L-GQDs showed comparable values at the two time points, NH_2_-GQDs and COOH-GQDs increased two times (Figure 5A,C). On the other hand, blue light treatment determined a significant increase in ROS production at both 60 min (approximately 5% for L-GQDs, 15% for NH_2_-GQDs, 25% for COOH-GQDs) following irradiation and 4 h later (approximately 25% for L-GQDs and NH_2_-GQDs, 50% for COOH-GQDs) (Figure 5B,D).

**Figure 6:**
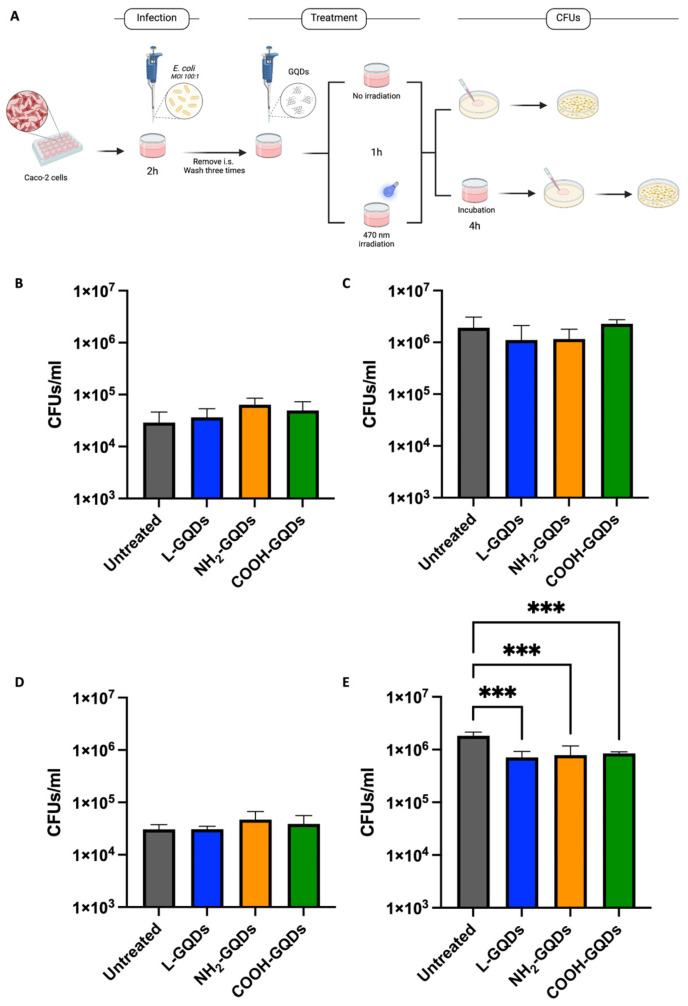
Graphene Quantum Dots reduce *E. coli* persistence in human epithelial cells. Schematic representation of the experimental model used to assess Graphene Quantum Dots (GQDs) antibacterial activity on human colorectal adenocarcinoma (Caco-2) cells infected with a suspension of *E. coli* (**A**). Caco-2 were infected with *E. coli* (MOI = 100:1) for two hours before removing infection solution and treating with 200 µg/mL GQDs (L-GQDs, NH_2_-GQDs and COOH-GQDs) and irradiated or not at 470 nm for 60 min. Colony forming units (CFUs) were evaluated at 1 (**B**,**D**) and 4 h (**C**,**E**). CFUs/mL were reported in log10 scale as bar plots. (*** *p* value < 0.001).

Caco-2 were finally infected with *E. coli* (MOI 100:1) for two hours before removing infection solution and administrating GQDs at final concentration of 200 µg/mL. As described in Figure 6A, Caco-2 were then exposed or not to 470 nm wavelength blue light for 60 min and then incubated in standard atmosphere conditions for additional 4 h when CFUs were assessed.

Results obtained from the CFUs count indicated that none of the GQDs were able to reduce the microbial burden in untreated cells (Figure 6B) or following 60 min of irradiation (Figure 6D). Otherwise, our data showed that after 4 h of incubation, Caco-2 blue light stimulated accounted a slight, but significant, reducing of *E. coli* burden compared to untreated or not stimulated cells (Figure 6C,E). Log_10_ differences between irradiated and not irradiated samples were also summarized in Supplementary Table S1.

**Figure 7:**
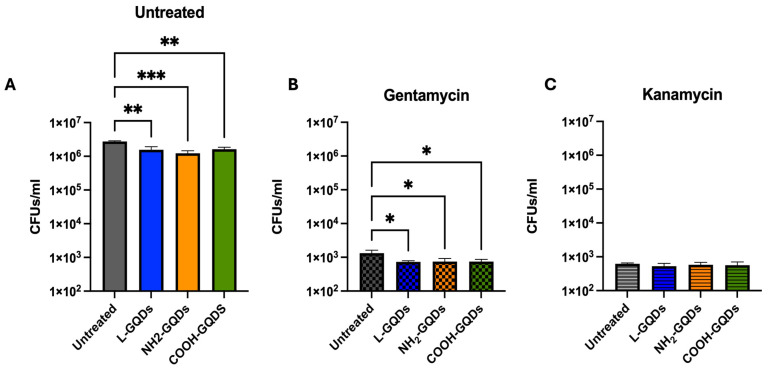
Graphene Quantum Dot drugs exhibited notable antibacterial effects during *E. coli* infection on the human colorectal adenocarcinoma cell line. Caco-2 cells were infected with a suspension of *E. coli* (MOI = 100:1). Two hours after infection, cells were washed and treated with 200 µg/mL GQDs (L-GQDs, NH_2_-GQDs and COOH-GQDs) and stimulated with blue light. One hour later, the medium was removed and new fresh medium containing gentamycin (**B**) or kanamycin (**C**) at minimal inhibitory concentrations (MICs) was added, except for control conditions (**A**), where only new fresh medium was added. CFUs were evaluated 4 h post treatment and represented in log10 scale. (* *p* value < 0.05; ** *p* value < 0.01; *** *p* value < 0.001).

To further investigate GQD activity directly on extracellular bacteria or indirectly on bacteria inside the cells, we repeated the previously described experiment introducing an additional antibiotic treatment together with nanomaterials administration (Figure 7). Caco-2 cells were infected with an *E. coli* suspension (MOI = 100:1), washed two hours later, treated with 200 µg/mL of each GQDs and exposed to blue light. One hour later, fresh medium containing gentamicin or kanamycin at minimum inhibitory concentrations (MICs) was added, except for control conditions, where only fresh medium was added. CFUs were assessed four hours later. While the CFUs count of the untreated cells showed higher growth of *E. coli* and confirmed GDQs’ antibacterial activity (Figure 7A), gentamicin treated cells showed a similar trend even though with an evident decrease in bacterial burden (Figure 7B). Interestingly, kanamycin co-administration was observed to decrease CFUs in all conditions (Figure 7C). Taken together, these results suggest a promising activity of GQDs, when irradiated with blue light, that triggers ROS production and appears mostly active in the extracellular milieu.

## 3. Discussion

The increase in antibiotic resistance represents a worldwide health issue, requiring the investigation of new treatment approaches. GQDs have showed enormous potential as antimicrobials because of notable chemical, physical, and biological features, rather than general advantages over traditional QDs robust fluorescence, photobleaching resistance, exceptional solubility and biocompatibility [17,22,29]. A number of studies have drawn attention to their antimicrobial properties against a wide range of microorganisms, including antibiotic-resistant bacteria and fungi [30]. Their exceptional surface area-to-volume ratio and compact size enable them to effectively interact with bacterial cells and due to diverse surface functional groups interact with microbes, enhancing cellular penetration and molecular targeting [3,31].

Unfortunately, these applications remain still elusive and controversial lacking precise and common experimental methodologies [23,32]. Despite of the facile synthesis compared to other nanomaterials, obtaining high-quality products is difficult because of the existing synthesis that generally produces small-scale amounts of GQDs which have a wide size distribution [20,31]. This inconsistency hinders the comparison of results across diverse studies and limits definitive conclusions about their efficacy.

Since the size, shape and functionalization have a massive impact on GQDs physicochemical properties, we used previously characterized GQDs [3] on *E. coli* as a model of Gram-negative bacterial pathogens.

Our results highlighted GQDs’ antimicrobial activity against *E. coli* when these nanomaterials were exposed to blue light (470 nm). A reduction of up to 2 logs in the CFUs count was measured 4 h following blue light exposure and up to 3 logs 24 h later. Interestingly, we found no differences dependent on GQD functionalization.

Our results appear in line with Ristic and colleagues, who observed a significant decrease in the *E. coli* and *S. aureus* bacterial burden after treatment with 200 µg/mL non-functionalized GQDs 4 h later [14]. Similarly, Rojas-Andrade and colleagues noticed antibacterial effects mediated by GQDs or reduced GQDs (rGQDs) against *S. epidermidis*. Note that the latter study assessed antimicrobial activity by measuring CFUs up to 18 h: GQDs and rGQDs were stimulated with a different wavelength light (395–400 nm) for 3 min before plating [21]. Although no light stimulation was employed, the GQDs-TiO2 combination lowered *S. mutans* burden after 24 h of incubation [33]. Stanković and colleagues firstly proposed blue light stimulation of hydrophobic carbon quantum dots that achieved significant antibacterial efficacy against *E. coli* and *S. aureus* [34]. In other words, GQDs appear to be promising antimicrobial compounds, but methodological differences in terms of exposure time, light stimulation and bacterial strains lead to difficulties in achieving complete knowledge of GQDs’ antimicrobial activity. In this scenario, our data support the evidence reported in previous studies, which were focused on GQDs short-term effects, typically within a few hours of incubation or few minutes of light stimulation, potentially overlooking the long-term dynamics of bacterial regrowth or resistance development. We assessed antimicrobial activity 24 h following treatment and stimulation underlying how light exposure appears to be the key factor to determining GQDs’ efficacy. Long-term observation was important to assessing GQDs’ multiple functional mechanisms suggested to promote antimicrobial activity.

SEM analysis clearly revealed that GQDs were not able to induce substantial morphological damage to bacterial cells, suggesting that they impact prevalently on the bacterial metabolism. This finding strengthens previous observations indicating GQDs’ antibacterial effects through mechanisms that do not heavily disrupt the physical integrity of bacterial cells. GQDs’ antibacterial effect is largely attributed to ROS generation: through the interaction with bacterial cells, they are able to various ROS such as singlet oxygen, hydroxyl radicals, and superoxide anions [35,36]. These ROS are highly reactive and can cause oxidative damage to essential biomolecules within the bacterial cells, including DNA, proteins, and lipids [37,38]. Furthermore, ROS production significantly increased when GQDs were stimulated with blue light directly impairing *E. coli* growth. Even though, aminated GQDs has been described as the powerful ROS stimulator, we note comparable ROS production of COOH- and NH_2_-QGDs [39]. The impairment of *E. coli* metabolism is in line with the results of Ouyang and colleagues, who explained GQDs’ antimicrobial activity with the increase in nitrogen-fixing gene expression [40]. This activity may be translated in an engulfment or alteration of the bacterial growth, much more than and effective damage, that may conduce to an early stationary phase [41].

These findings highlight the complexity of GQDs’ interactions, maybe due to different functionalization and experimental settings, and underscore the necessity for further experimental standardization to elucidate their precise mechanisms of action. At the same time, a better understanding of these mechanisms may result in dynamic applications of these nanomaterials.

It is important mention to mention that blue light that has emerged as a potent antimicrobial adjuvant due to its ability to induce photodynamic effects that generate ROS [42]. This wavelength is particularly effective in aPDT because it can penetrate tissues significantly, making it useful for both superficial and deeper infections [43]. Importantly, much research has shown that blue light (at 470 nm) can effectively disrupt the cell walls and membranes of various bacteria [42,44,45]. Additionally, blue light inhibits biofilm formation, a major challenge in treating chronic infections, by penetrating the biofilm matrix and killing embedded bacteria [46,47]. The blue light-GQDs combination may enhance the excitation of endogenous photosensitizers within microbial cells, producing several ROS species [48,49].

Moreover, the low toxicity of blue light to human cells, compared to ultraviolet light, makes it a safer option for clinical applications [50,51,52]. These findings are coherent with our study, since we observed that Caco-2 cells did not show cytotoxicity when exposed to each GQD or blue light, either alone or in combination. Nevertheless, the combination of blue light and GQDs has been explored for the treatment of specific cancers, with the objective of eliminating tumor cells [9,53,54]. Given that ROS production has been described in eukaryotic cells for cancer treatment and these were also generated when macrophages were treated, graphene oxide was used [26]. We measured their production in our Caco-2-based in vitro model. Indeed, the generation of ROS was observed when Caco-2 cells were treated with GQDs and were stimulated by blue light. The major activity measured when GQDs were used in combination with gentamicin suggested that these nanomaterials prompt for antibiotic internalization. Gentamicin, which is known to not efficiently penetrate into the eukaryotic cell membrane, after GQDs and blue light stimulation significantly reduced bacterial burden [55].

This finding may be a key point in the defense against bacterial pathogens because ROS are normally produced by cells that are involved in the host-defense response [56]. Moreover, few studies have investigated the antibacterial activity of GQDs in in vitro models [3,57] so further investigations, also based on more complex and multicellular models and antibiotic administrations, will be needed to ascertain the clinical role of the GQDs and blue light combination, evaluating the possible ROS-mediated cellular signaling and its involvement in host immunity regulation.

## 4. Methods

### 4.1. Bacterial Manipulation

*Escherichia coli (E. coli)* was grown in Luria–Bertani (LB) broth medium (Sigma-Aldrich, Saint Louis, MO, USA) at 37 °C overnight. Bacterial culture was then harvested, and 20% sterile pure glycerol (Carlo Erba Reagents, Milano, Italy) was added. Aliquots of the suspension were finally stored at −80 °C and then assessed in terms of colony forming units (CFUs). All experiments that involved *E. coli* manipulation were performed in Biosafety level 2 laboratory in the Institute of Microbiology of Fondazione Policlinico Universitario A. Gemelli—IRCCS.

### 4.2. Graphene Quantum Dots Features

GQDs were previously characterized encompassing optical density, fluorescence intensity, dynamic light scattering (DLS), zeta potential, and attenuated total reflection-Fourier transform infrared spectroscopy (FITR) [3]. L-GQDs and COOH-GQDs exhibited an absorption shoulder at 350 nm, whereas NH2-GQDs demonstrated a shifted absorption peak toward 400 nm. Fluorescence intensity spectra were recorded by exciting GQDs from 250 to 520 nm and measuring emission from 300 to 700 nm (10 nm step size), normalizing the highest recorded emission for each GQD. L-GQDs, NH2-GQDs and COOH-GQDs showed emission peaks at 380 nm (excitation 330 nm), 450 nm (excitation at 380 nm) and 450 nm (excitation at 330 nm), respectively. DLS and zeta potential analyses indicated a hydrodynamic radius peaked below 10 nm and a surface charge of −47.3  ±  5.95 mV (L-GQDs), −21.2  ±  2.93 mV (COOH-GQDs) and −3.2  ±  1.72 mV (NH2-GQDs). FITR analysis highlighted that L-GQDs had two bands in the fingerprint region typical of the C=O bond; NH2-GQDs displayed a broad band at approximately 3000 cm^−1^, corresponding to the N–H stretching vibration of amine groups and a broader band at approximately 1400 cm^−1^ indicating C-N absorption. Finally, characteristic frequencies of the carboxylic groups, due to a broad band between 3500 and 2500 cm^−1^ for the O-H stretch and a prominent band at 1705 cm^−1^ for the C=O stretch, was observed in COOH-GQDs.

### 4.3. In Vitro Antimicrobial Assay

A suspension of *E. coli* (5 × 10^5^ CFUs/mL) was incubated alone or with Blue Luminescent GQDs (L-GQDs), Aminated GQDs (NH_2_-GQDs), or Carboxylated GQDs (COOH-GQDs) (ACS Material) at the final concentration of 200 µg/mL to assess direct activity of GQDs in LB medium at 37 °C [3,14,58,59]. Bacterial solutions were also exposed to blue light (470 nm) irradiation for 15 min (min), 30 min and 1 h (h) without mechanical agitation before 4 h and 24 h incubation in standard atmosphere conditions at 200 rpm. Blue light showed the following features: 5 VDC (operating voltage range 2.5~6 VDC), 155 mA–179 mA, 0.78–0.90 watts. Colony forming units (CFUs) were determined at time 0 (infection solution), 15 min, 30 min and 1, 4 and 24 h by plating serial dilutions (1:10) of the bacterial suspensions on LB agar medium (Sigma-Aldrich, Saint Louis USA). CFUs/mL were then obtained by multiplying the number of CFUs (colonies of *E. coli*) on agar plates by the appropriate dilution factor (Figure 1A).

### 4.4. Scanning Electron Microscopy (SEM)

SEM was performed to evaluate the macroscopic effect of GQDs on a 5 × 10^5^ *E. coli* suspension. The *E. coli* suspension was treated with 200 µg/mL of each GQD (L-GQDs, NH_2_-GQDs, COOH-GQDs) followed by 60 min of blue light irradiation. Treated and control conditions were spotted onto polyurethane disks, followed by fixation in 2.5% glutaraldehyde and dehydration in ethanol [25].

Images were taken using a SEM Supra 25 (https://speciation.net/Database/Instruments/Carl-Zeiss-AG/Supra-25-;i665 accessed on 21 July 2024, Zeiss, Oberkochen, Germany). Images were taken at 15 K magnification (scale bar is indicated on each image). Images were analyzed using FIJI software (https://fiji.sc/ accessed on 21 July 2024, National Institute of Health, Bethesda, MD, USA).

### 4.5. Graphene Quantum Dots Effect on E. coli Metabolic Activity

The metabolic impact of GQDs on *E. coli* was investigated utilizing the MTS assay (Abcam, Cambridge, UK) in a controlled experimental setup. A bacterial suspension with a concentration of 5 × 10^5^ CFU/mL was inoculated into a 96-well plate with flat bottom (Corning, New York, NY, USA). Subsequently, 200 µg/mL of functionalized GQDs (L-GQDs, NH_2_-GQDs, and COOH-GQDs) were introduced to the bacterial cultures. The experimental conditions included exposure to blue light for a duration of 60 min, as well as a control condition without blue light exposure. To establish baseline metabolic activity, 2% Triton X-100 (Sigma-Aldrich) served as a positive control for maximum metabolic inhibition, while untreated bacterial suspensions were used as negative controls. Post incubation, the MTS assay was conducted, and the metabolic activity was quantified by measuring absorbance at 490 nm using a spectrophotometer [27,60]. All experiments were performed in triplicate to ensure reproducibility.

### 4.6. Assessment of ROS Generation in E. coli Culture

*E. coli* was cultured in LB broth medium at 37 °C with shaking at 200 rpm overnight. The cells were harvested by centrifugation at 4000 rpm for 10 min, washed twice with phosphate-buffered saline (PBS, pH 7.4), and resuspended in PBS to a final concentration of 5 × 10^5^ cells/mL. Bacterial cells were incubated alone or with Blue Luminescent GQDs (L-GQDs), Aminated GQDs (NH_2_-GQDs), or Carboxylated GQDs (COOH-GQDs) (ACS Material) at the final concentration of 200 µg/mL and stimulated with blue light (470 nm) for 60 min or not. Intracellular reactive oxygen species (ROS) levels were measured using the DCFDA/H2DCFDA—Cellular ROS Assay Kit (Abcam, Cambridge, UK) [60]. *E. coli* cells were incubated with 20 µM of 2′,7′-dichlorofluorescin diacetate (DCFDA) at 37 °C for 30 min in the dark. Following incubation, cells were washed twice with H_2_O to remove excess DCFDA. The treated cells were then aliquoted into a 96-well black-bottom microplate (Agilent, Santa Clara, CA, USA), with 100 µL of cell suspension per well. The fluorescence intensity, indicative of ROS levels, was measured using the Cytation 5 Cell Imaging Multi-Mode Reader (BioTek Instruments, Winooski, VT, USA) with excitation and emission wavelengths set at 485 nm and 535 nm, respectively. All experiments were performed in triplicate to ensure reproducibility. Fluorescence data were analyzed using Gen5 software (BioTek Instruments).

### 4.7. Caco-2 Cells Culture and Infection

Caco-2 cells (ATCC, Manassas, VA, USA; Accession Number: CVCL_0025) were cultured in DMEM (Euroclone, Milano, Italy) supplemented with 20% inactivated FBS (Euroclone, Milano, Italy), 1% L-glutamine (Euroclone, Milano, Italy) and 1% streptomycin–penicillin (Euroclone, Milano, Italy) and were incubated at 37 °C and 5% CO_2_. Adherent cells were washed with sterile warm PBS (Euroclone, Milano, Italy) and removed for experiments by using 1× trypsin in PBS (Euroclone, Milano, Italy). Cells were counted and re-suspended in DMEM supplemented with 2% FBS and 1% L-glutamine. Finally, cells were seeded in sterile 48 well plates (Euroclone, Milano, Italy) at a concentration of 5 × 10^5^ cells/mL and incubated overnight until infection or treatment. Caco-2 cells were infected with *E. coli*, with multiplicity of infection (MOI) of 100 (100 bacterium to 1 cell), resuspended in the cell culture medium. One hour post infection, infected cells were washed with sterile warm PBS to remove extracellular bacteria and treated with irradiated or non-irradiated GDQs for 1 h. Cells were incubated in standard atmosphere conditions for 24 h when CFUs were assessed by harvesting the cell monolayer with sterile 0.1 mL of sterile 0.05% Triton X-100 (Sigma-Aldrich). Serial dilutions in (1:10) of the cellular lysate were carried out before plating on LB agar plates. Plates were then incubated at 37 °C overnight (Figure 5A). CFUs/mL were obtained by multiplying the number of CFUs (colonies of *E. coli*) on agar plates by the appropriate dilution factor and reported as log CFUs/10^6^ cells. To investigate the effects of GQDs on bacteria located outside cells as well as those within cells, the initial experiment was conducted again, incorporating an additional antibiotic treatment along with the nanomaterials. Gentamicin (Euroclone, Milano, Italy) and kanamycin (Sigma-Aldrich, USA) were administrated at minimal inhibitory concentrations (MICs), carrying out the experiment as described previously (Figure 6).

### 4.8. Evaluation of Cytotoxicity on Caco-2 Cells

To evaluate the effects of GQDs on cell viability, Caco-2 cells (ATCC, Manassas, VA, USA; Accession Number: CVCL_0025) were cultured and subsequently seeded at a density of 5 × 10^5^ cells per mL in 96-well plates (Corning, New York, NY, USA). The Caco-2 cells were incubated overnight under standard conditions (37 °C, 5% CO_2_) to ensure proper cell adherence and stabilization. Following this, the cells were treated with 200 µg/mL of three different GQD formulations (L-GQDs, NH_2-_GQDs, and COOH-GQDs) with or without exposure to blue light at a wavelength of 470 nm. Untreated cells were used as a negative control, while cells exposed to 2% Triton X-100 (Sigma-Aldrich) served as a positive control to induce maximum cytotoxicity. The MTS Cell Proliferation Assay (Abcam, UK) was then employed to assess the metabolic activity of the Caco-2 cells post treatment [3]. One hour after incubation with the GQDs, MTS reagent was added to each well and the plates were incubated for an additional 4 h at 37 °C. The absorbance was measured at 490 nm to quantify nanomaterials cytotoxicity.

### 4.9. Assessment of ROS Generation in Human Epithelial Cells

To assess ROS generation, human epithelial colorectal adenocarcinoma cells (Caco-2) (ATCC, Virginia, USA; Accession Number: CVCL_0025) were cultured in Dulbecco’s modified Eagle’s medium (DMEM) supplemented with 20% fetal bovine serum (FBS), 1% L-glutamine (Euroclone. Italy) and 1% penicillin-streptomycin (Euroclone, Italy) at 37 °C in a humidified atmosphere with 5% CO₂. The cells were seeded into 96-well black-bottom microplates (Agilent, USA) at a density of 1.2 × 10^6^ cells/well and allowed to adhere overnight. After 24 h, cells were washed twice and the medium was replaced with fresh RPMI 1640 (Euroclone, Italy) containing Blue Luminescent GQDs (L-GQDs), Aminated GQDs (NH₂-GQDs), or Carboxylated GQDs (COOH-GQDs) (ACS Material) at a final concentration of 200 µg/mL. The cells were either exposed to blue light (470 nm) for 60 min or kept in the dark as a control. Intracellular reactive oxygen species (ROS) levels were assessed using the DCFDA/H2DCFDA—Cellular ROS Assay Kit (Abcam, UK) [26]. Caco-2 cells were incubated with 20 µM of 2′,7′-dichlorofluorescin diacetate (DCFDA) at 37 °C for 45 min in the dark. Post incubation, cells were washed twice with H_2_O to remove excess DCFDA. Fluorescence intensity, reflecting ROS levels, was measured using the Cytation 5 Cell Imaging Multi-Mode Reader (BioTek Instruments, Winooski, VT, USA) with excitation and emission wavelengths set at 485 nm and 535 nm, respectively. All experiments were conducted in triplicate to ensure data reliability. Fluorescence readings were analyzed using Gen5 software (BioTek Instruments).

### 4.10. Statistical Analysis

Data were collected and organized using Microsoft Excel software version 16.69.1 and were analyzed by using GraphPad Prism software version 9.0.0 (GraphPad software) and R v4.0.2. All experiments were performed in scientific duplicates and technical triplicates. All data were expressed as mean plus SD and analyzed by two-way ANOVA followed by the appropriate correction.

## 5. Conclusions

Our study emphasized the antibacterial properties of functionalized GQDs against *E. coli*, particularly when stimulated with blue light. Furthermore, we pointed out the use of standardized models based on axenic cultures or in vitro infection models to ensure the reliability and reproducibility of these findings across studies, and previously characterized GQDs were employed. In summary, GQD activity was blue light mediated and time dependent. Although no evident effect was detected on E. coli morphology, GQDs impair bacterial metabolism and stimulate ROS production.

## Data Availability

The original contributions presented in this study are included in this article, and further inquiries can be directed to the corresponding author.

## Supplementary Materials

The following supporting information can be downloaded at: https://www.mdpi.com/article/10.3390/ijms25158033/s1.
