# Supplementary material for: Exploration of the Graphene Quantum Dots-Blue Light Combination: A Promising Treatment against Bacterial Infection
Source: Int J Mol Sci. 2024 Jul 23;25(15):8033. doi: 10.3390/ijms25158033 (PMC11312127; doi:10.3390/ijms25158033)
Supplement: Supplementary file 1 [file ijms-25-08033-s001.zip › ijms-3091871-supplementary.pdf]

Axenic culture	Time points	Untreated	L-GQDs	NH_2-GQDs	COOH-GQDs
15 min irradiation	15 min	-0.50	-0.68	-0.34	-0.39
	4 hours	-0.85	-0.97	-1.31	-1
	24 hours	-0.73	-2.15	-1.80	-1
30 min irradiation	30 min	-0.50	-0.72	-0.75	-0.45
	4 hours	-0.79	-0.78	-1.04	-1.45
	24 hours	-0.69	-2.40	-2.15	-1.72
60 min irradiation	60 min	-0.34	-1.2	-0.58	-0.59
	4 hours	-1.66	-3.90	-3.06	-4
	24 hours	-1.95	-3.05	-2.47	-1.48

In vitro infection	Time points	Untreated	L-GQDs	NH_2-GQDs	COOH-GQDs
60 min irradiation	60 min	-0.25	-0.24	-0.48	-0.27
	4 hours	-0.16	-0.64	-0.20	-0.50

Supplementary Table S1. Log10 differences between CFUs values of non-stimulated samples and blue light irradiated samples. Both not-treated (Untreated) and GQDs (L-GQDs, NH_2-GQDs and COOH-GQDs) treated samples were represented. For axenic cultures were reported data of 15, 30 and 60 minutes of irradiation and CFUs measured at different time points, while for Caco-2 infected cells were reported data after 60 minutes of irradiation and CFUs measurement at 60 minutes and 4 hours.
